# Genetic Architecture of Conspicuous Red Ornaments in Female Threespine Stickleback

**DOI:** 10.1534/g3.115.024505

**Published:** 2015-12-29

**Authors:** Lengxob Yong, Catherine L. Peichel, Jeffrey S. McKinnon

**Affiliations:** * Department of Biology, East Carolina University, Greenville, North Carolina 27858; † Division of Basic Sciences, Fred Hutchinson Cancer Research Center, Seattle, Washington 98109; ‡ Division of Human Biology, Fred Hutchinson Cancer Research Center, Seattle, Washington 98109

**Keywords:** sexual dimorphism, QTL mapping, *Gasterosteus aculeatus*, red nuptial coloration, female ornaments

## Abstract

Explaining the presence of conspicuous female ornaments that take the form of male-typical traits has been a longstanding challenge in evolutionary biology. Such female ornaments have been proposed to evolve via both adaptive and nonadaptive evolutionary processes. Determining the genetic underpinnings of female ornaments is important for elucidating the mechanisms by which such female traits arise and persist in natural populations, but detailed information about their genetic basis is still scarce. In this study, we investigated the genetic architecture of two ornaments, the orange-red throat and pelvic spine, in the threespine stickleback (*Gasterosteus aculeatus*). Throat coloration is male-specific in ancestral marine populations but has evolved in females in some derived stream populations, whereas sexual dimorphism in pelvic spine coloration is variable among populations. We find that ornaments share a common genetic architecture between the sexes. At least three independent genomic regions contribute to red throat coloration, and harbor candidate genes related to pigment production and pigment cell differentiation. One of these regions is also associated with spine coloration, indicating that both ornaments might be mediated partly via pleiotropic genetic mechanisms.

Sexual selection theory commonly predicts that males should be the most ornamented sex; however, female ornaments are now known to be widespread, and are the subject of increased research efforts (Darwin 1871; Andersson 1994; Kraaijeveld *et al.* 2007; Kraaijeveld 2014). Interest in the causes and functions of female ornaments has prompted studies that have often attributed their evolution to selective pressures similar to those responsible for male ornament evolution, *i.e.*, male choice and female–female competition (Andersson 1994; Kraaijeveld *et al.* 2007; Clutton-Brock 2009; Tobias *et al.* 2012). However, selection favoring female ornaments can also be weak or absent in some taxa, pointing to the potential involvement of nonadaptive processes (Muma and Weatherhead 1989; Cuervo *et al.* 1996; Nordeide 2002; Wright *et al.* 2015; Yong *et al.* 2015). Thus, no clear consensus has yet emerged on the evolutionary causes of female ornaments.

While behavioral and comparative studies have been informative, a thorough understanding of female ornament evolution also requires knowledge of the underlying genetic architecture. The genetic basis of female ornaments is particularly interesting because such ornaments are notably labile, in that they can be frequently lost and regained on a phylogeny (Omland 1997; Cardoso and Mota 2010; Kraaijeveld 2014). The observation that some female ornaments can evolve and persist as rudimentary versions of male-typical traits suggests that shared genetic mechanisms between the sexes might contribute to ornament expression (Lande 1980; Amundsen 2000; Clutton-Brock 2009; Potti and Canal 2011).

Although the genetic basis, and particularly the molecular genetics, of female ornaments is still poorly understood (Chenoweth and McGuigan 2010; Kraaijeveld 2014), some progress has been made. In the fowl (*Gallus gallus*) and zebrafinch (*Taeniopygia guttata*), female comb ornaments and beak redness seem to be under the control of a few loci of moderate effect, pointing to a relatively simple genetic basis (Wright *et al.* 2008; Schielzeth *et al.* 2012). The same loci are also detected in males, suggesting that the presence of the female ornaments might in part result from a shared genetic architecture.

The threespine stickleback fish (*Gasterosteus aculeatus*) has offered exceptional opportunities for detailed genetic investigation of evolutionary diversification, including studies of secondary sexual characters (Peichel *et al.* 2001; Albert *et al.* 2008; Kitano *et al.* 2009; Malek *et al.* 2012). Indeed, sticklebacks possess a well-studied secondary sexual trait, carotenoid-based orange-red throat coloration (Figure 1A), which was long thought to be exclusive to males, and is important in male–male competition and female choice (Bakker and Milinski 1993). Recent work has shown that females also exhibit the male-typical red throat in some freshwater populations that are derived from ancestral marine and anadromous forms (von Hippel 1999; McKinnon *et al.* 2000; Yong *et al.* 2013; Figure 1B).

**Figure 1:**
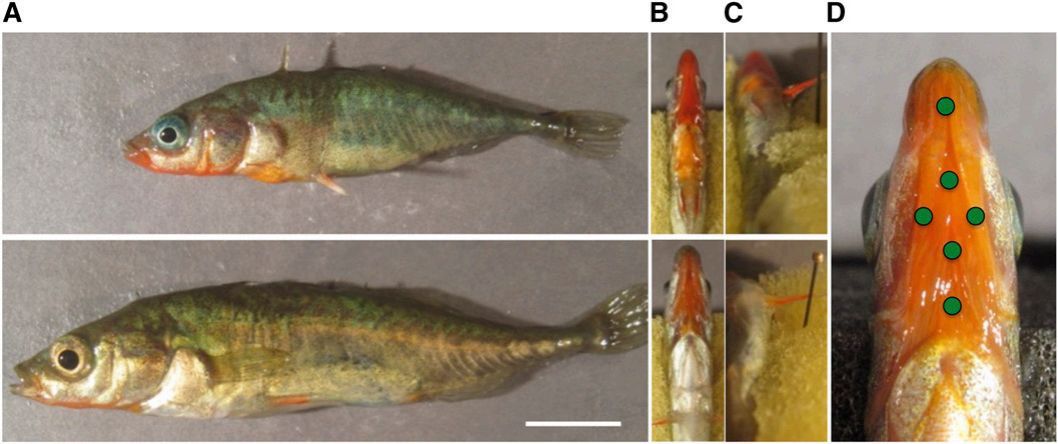
Digital photographs of MAT male (top) and female (bottom) sticklebacks. (A) full lateral view; (B) ventral view of red throat; (C) ventral view of red spine; (D) throat image with landmarks (green point) from which spectrometric measures were taken. White scale bar = 1 cm.

Another interesting, and presumably carotenoid-based, color trait in sticklebacks is red pelvic spine coloration (Nordeide 2002; Hodgson *et al.* 2013; Yong *et al.* 2013; Amundsen *et al.* 2015; Figure 1C). Although often observed, spine coloration seems to vary across stickleback populations, such that it is not present in all populations, and its adaptive function remains unclear (Nordeide 2002; Amundsen *et al.* 2015; L. Yong, unpublished data). Spine coloration is likely an ancestral trait in the Gasterosteidae lineage (McLennan 1996), and is generally more intense in Pacific marine/anadromous populations, compared to freshwater populations (Yong *et al.* 2013). The degree of sexual dichromatism in spine coloration varies, but it is somewhat sexually dimorphic in some freshwater populations (Yong *et al.* 2013; Amundsen *et al.* 2015). While little is known about why spine coloration is less intense in freshwater populations, several factors have been proposed, including potential trade-offs between different color traits and carotenoid limitation (Nordeide *et al.* 2006; Svensson and Wong 2011). To date, no studies have been conducted of the molecular genetics of variation in spine coloration, although the genetic basis of pelvic spine presence or absence is well understood (Cresko *et al.* 2004; Shapiro *et al.* 2004; Coyle *et al.* 2007; Chan *et al.* 2010; Shikano *et al.* 2013).

We have previously shown that the red color intensity of the spine is correlated with that of the throat in wild populations with red-throated females (Yong *et al.* 2013). However, it seems that both traits in females might have limited adaptive functions with regard to sexual selection (Nordeide 2002; Wright *et al.* 2015; Yong *et al.* 2015). For instance, female red throats provide no social advantage in the context of either intra or intersexual interactions (Wright *et al.* 2015; Yong *et al.* 2015). Contrary to the view that producing multiple ornaments is costly (Svensson and Wong 2011), a correlation between the two colorful ornaments may indicate limited internal tradeoffs, and instead suggests that the same genetic variants might influence the development and evolution of the two color patches.

In the present study, we investigated the genetic basis of orange-red throat and spine color (hereafter red throat or spine chroma) in female, as well as in male, threespine stickleback using quantitative trait loci (QTL) mapping. First, we characterized the number and location of genomic regions underlying each color trait, and asked to what extent the genetic architecture is shared between males and females. The detection of QTL in the same genomic regions in males and females would suggest that shared genetic mechanisms might underlie the expression of the ornaments in both sexes. We then asked whether common QTL might control both throat and spine color.

## Materials and Methods

### Fish collection, husbandry, and crosses

Sticklebacks used for crosses were obtained from two different freshwater sources. Females with red throats were collected from Matadero creek (MAT) in California (37.393° N, 122.162° W) using seines and dipnets (Yong *et al.* 2013). We specifically focus on the MAT population because those females display red coloration readily and intensely under laboratory conditions (L. Yong, unpublished data), making them suitable for genetic linkage mapping. MAT female sticklebacks were crossed with lab-raised Paxton (PX) limnetic male sticklebacks (49.703° N, 124.522° W), a population in which females have never been observed to possess red throat coloration. While MAT sticklebacks express red bright pelvic spines, PX limnetic males and females tend to have very little color on theirs (J. Boughman, personal communication). Fish were maintained in 102 liter tanks filled with aerated and purified water (3 ppt with Instant Ocean salt), and under a summer photoperiod (16-hr light: 8-hr dark) using natural spectrum-mimicking fluorescent light (Lumichrome Full Spectrum, Plus, Lumiram Electric, Co. Larchmont, NY) at 17–20°. All fish were fed a mixture of brine shrimp (*Artemia*) and bloodworms (chironomidae) twice a day. All animal work and experimental procedures conformed with ECU’s Institutional Animal Care and Use Committee (AUP #224a).

The experimental population involved a backcross, a favorable approach to overcome the somewhat weak expression of female throat coloration previously observed in a preliminary intercross F2 design (J. McKinnon, personal observation). A single adult MAT female was first crossed *in vitro* to a PX male to generate an F1 family. At sexual maturity (∼1 year old), three adult F1 males were backcrossed to a single MAT female to generate three half-sib families (*n* = 130–200 per family). All backcross offspring were reared in controlled conditions comparable to those described above. Because stickleback throat coloration can be influenced by carotenoids available in the food source (Pike *et al.* 2011), all fry were fed a consistent diet. Hatchlings were fed fresh live brine shrimp nauplii for the first 3 months, and then gradually transitioned to a slurry of minced frozen bloodworms and brine shrimp as they matured into juveniles and adult fish. At 6 months of age, fish were separated among 102 liter tanks (*n* = 25–30 fish per tank) to ensure adequate growth rates. To minimize the potential effects of background on color, all sides except for the front of each tank were covered with brown paper. Light and temperature cycles were carefully controlled, emulating natural conditions, such that all experimental fish experienced two winter-like (8-hr light: 16-hr dark; 10°; 4 months) and summer-like (16-hr light: 8-hr dark; 20°; 4 months) light cycles and temperatures. Both temperature and light were incrementally adjusted between the seasonal cycles. All backcross fish were reared in the laboratory under these standardized conditions until 2 years of age, and then phenotyped.

### Male nesting stimulation

While the expression of the male throat coloration is generally heightened during the breeding season, it also varies with sexual context. For instance, male coloration is often more intense during the courting phase of the mating cycle (Bakker and Mundwiler 1994). Thus, for standardization and maximal expression of color, we measured the throat coloration of males after they had successfully nested and courted females.

Before introducing males to a nesting tank, they were first measured for red throat chroma (pre-nesting throat color) (see *Phenotyping* below). They were then placed in a 30-liter tank and provided with a plastic dish containing sand and sphagnum moss as nesting material. After males acclimated to their tank, they were presented with a gravid female enclosed in a UV transparent container for 10 min twice daily for 3 d to promote nesting and courting behaviors. Males typically began nesting behavior by digging in the sand, which occurred within 24 hr after introduction into their nesting tank. On the 4th day, males were presented with a gravid female for a final time, and allowed to court the female for 15 min. Thereafter, males were immediately netted and measured for throat color (post-nesting throat color). Males that had nested and courted females had significantly redder throat coloration than males that had not (*t*-test: *P* < 0.0001). For consistency, only backcross males that had nested and courted females were included in the final genetic mapping analyses (*n* = 148 out of 193). Unlike males, females exhibit no significant changes in throat coloration according to the reproductive cycle (Yong *et al.* 2013); backcross females were thus phenotyped without being subjected to similar treatments (*n* = 281).

### Phenotyping: throat and spine chroma

All fish, *e.g.*, grandparents, F1, and backcross individuals (193 males, 281 females), were measured for standard length (nearest 1 mm) using digital calipers and mass using a small balance. Fish were sexed based on distinct breeding color (*i.e.*, red nuptial throat associated with blue eyes in males), proxies of reproductive status (*i.e.*, gravid status for females), and with the *Idh* genetic marker (Peichel *et al.* 2004). Wild MAT fish, pure lab crosses of MAT fish, and F1 hybrids were also phenotyped as detailed below, and included in the phenotypic (but not genetic mapping) analyses for comparison.

Throats and left pelvic spines were photographed as detailed in Yong *et al.* (2013, figure 1A–C) under a natural light source (MR16 Solux Natural Daylight, Tailor Lightning Inc., Rochester NY). Using an Ocean Optics Maya spectrometer (Ocean Optics Inc., Dunedine, FL) and established protocols (Yong *et al.* 2013), the color reflectance of the fish throat was consistently measured at six spots (Figure 1D). The procedure for phenotyping took less than 3 min for each fish, and did not involve anesthetizing the fish. Fish were then killed in a lethal dose of MS-222 solution, and a caudal fin clip was collected for DNA extraction and genotyping.

To obtain the red color score for both throat and spine, we implemented the established protocol detailed in Yong *et al.* (2013). In brief, red throat color was quantified using a physiological model of stickleback vision to approximate stickleback-visual perception (Rush *et al.* 2003; Endler and Mielke 2005; Pike *et al.* 2011). Because the color intensity of the spine could not be measured using spectrometry due to its small patch size, it was assessed from standardized images with Adobe Photoshop CS3 (Adobe Systems, San Jose, CA). The pelvic spine was divided into eight equal sections, and the red, green and blue (RGB) values were obtained for each section. The red chroma from each segment was then estimated by calculating red intensity relative to the combined intensities of blue, red, and green (Yong *et al.* 2013). We have previously shown that this approach correlates significantly positively with spectrometric values (Yong *et al.* 2013). For each ornament, we then obtained a red color score (hereafter red throat chroma, and red spine chroma) by averaging across all sampled spots from which measurements were obtained. We have used the maximum intensity of chroma in our past studies (McKinnon *et al.* 2000; Yong *et al.* 2013, 2015; Wright *et al.* 2015), largely owing to limitations of our reflectance sampling; here, we used average chroma because our modified reflectance protocol captured color variation throughout the throat more systematically and comprehensively. We have found significant positive correlations between maximum and mean measures of chroma (and also for different methods, *e.g.*, Yong *et al.* 2013), but it is worth noting that, while they often produce similar results, this is not always the case as they measure slightly different aspects of color.

### Genotyping and linkage map

Both grandparents, the three F1 male and one MAT female parents, and 429 backcross offspring (148 nested males, 281 females) were genotyped using a custom designed single nucleotide polymorphism (SNP) array (Illumina, San Diego, CA) containing 768 SNP markers spanning the stickleback genome (Greenwood *et al.* 2011, 2013, 2015; Jones *et al.* 2012; Wark *et al.* 2012; Arnegard *et al.* 2014; Conte *et al.* 2015). Genomic DNA was first extracted from fin clips using the Qiagen DNAeasy Blood and Tissue kit (Qiagen Inc., Valencia, CA). DNA samples were diluted to 50–100 ng/μl, and genotyped at the Fred Hutchinson Cancer Research Center Genomics Shared Resource (Seattle, WA). SNP data were analyzed using Illumina GenomeStudio software. Among the SNP markers, we identified 229 informative markers (Supporting Information, Table S1) displaying allelic differences between the MAT and PX grandparents. Using a LOD threshold of 4.0 for all markers, 22 linkage groups (LG), representing the 21 stickleback chromosomes, were constructed using JoinMap 4.1 (Van Ooijen 2011).

### QTL mapping

We used standard interval mapping in R/qtl (Broman and Sen 2009) to identify genomic regions contributing to variation in throat and spine coloration in our backcross population. Red chroma scores of both throat and spine were log-transformed to improve normality. First, we mapped both throat and spine color by combining all females and nested males, *i.e.*, 429 backcross individuals, and including family and sex as covariates. Body size was also included as a covariate in the analyses, but later removed because its inclusion had little effect on the results (Broman and Sen 2009). Then, to test whether the same QTL persist and remained similar between the sexes, we mapped the same traits for males and females separately. We used permutation tests (*n* = 1000) to calculate the genome-wide significance thresholds (logarithm of odds or LOD score) for association between markers and red throat and spine chroma (α = 0.05). We calculated the 95% Bayesian credible intervals at each significant QTL position. Using the *fitqtl* and *refineqtl* functions in R/qtl, we determined QTL direction and effect size, as defined by the percentage of phenotypic variance explained (PVE), and refined the locations of our QTL (Broman and Sen 2009). Epistasis and additivity among QTL pairs in the whole dataset were determined using the *scantwo* function. As recommended (Broman and Sen 2009), we used a conservative LOD threshold of 4.7 for significant epistatic and additive interactions. To test whether all significant QTL acted additively, we also examined the relationship between the sum of MAT alleles across significant QTL and red throat chroma. The X-chromosome was omitted from the QTL analysis because all X chromosomes in this cross were from the MAT population due to the lack of recombination between the X and the Y chromosomes (Peichel *et al.* 2004) and our backcross experimental design. Thus, in this cross we were unable to map X-linked genetic differences between the MAT and PX populations. All genotype and phenotype data for the backcrosses are provided in File S1.

### Screening of candidate genes underlying ornament coloration

To identify potential candidate genes within the QTL, we searched for candidate genes located within the 95% Bayesian credible intervals of the QTL using the Ensembl release 78 stickleback genome database (BROADS1; ensembl.org/Gasterosteus_aculeatus/Info/Index).

### Statistics

Statistical analyses were conducted in the R statistical environment (version 3.1, R Development Group, http://www.r-project.org). Linear mixed models (*nlme* package in R) were used to test for differences in throat and spine chroma using sex and cross type (*i.e.*, pure MAT, F1 hybrids, and backcross individuals) as fixed effects, and family as a random effect (Pinheiro *et al.* 2015). Because males in both the pure crosses and F1 hybrids were non-nesting males, all backcross males at the non-nesting stage (*n* = 193) were included for comparing throat and spine coloration. *Post hoc* pairwise comparisons were subsequently conducted, and a false discovery rate was used to control for multiple comparisons between groups (Verhoeven *et al.* 2005). Corrected *P*-values from the comparisons within ANOVAs are reported.

### Data availability

## Results

### Red throat and spine coloration are correlated with each other in the backcross

**Figure 2:**
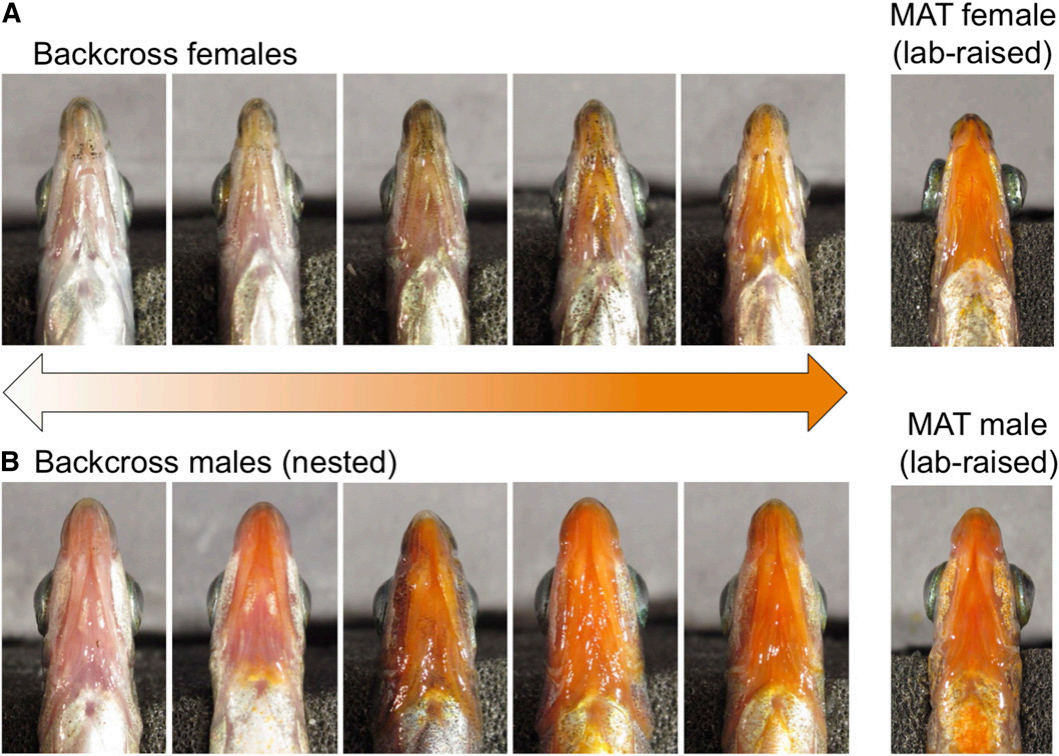
Variation in throat chroma in the backcross females (A) and males (B) in comparison to laboratory-raised MAT male and female.

**Figure 3:**
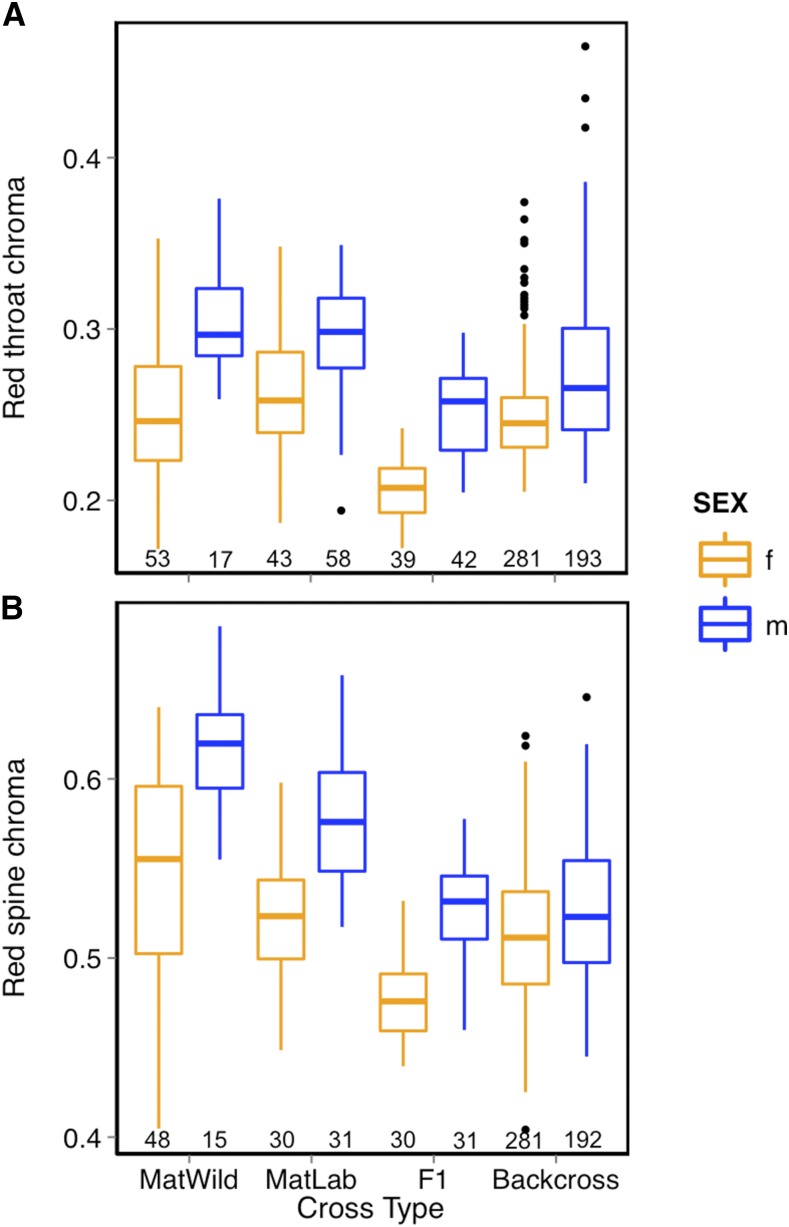
Sex-specific differences in (A) red throat and (B) spine chroma between cross types. Dots represent outliers. Numbers of individuals in each category are indicated at the bottom. Backcross, Backcross sticklebacks; F1, F1 hybrids; f, female; m, male; MatLab, lab-raised Matadero; MatWild, wild-caught Matadero.

To identify the genetic contributions to ornaments, we quantified throat and pelvic spine red chroma in both wild and laboratory-reared MAT sticklebacks, as well as in F1 and backcross hybrids. The recapitulation of the red throat and spine coloration in the laboratory-reared pure crosses suggests that both ornaments have a genetic component (Figure 2). Laboratory-raised MAT (MatLab) sticklebacks showed similar red throat and spine chroma to wild MAT sticklebacks (MatWild) (throat: cross type: F_1,2_ = 0.127, *P* = 0.755; sex: F_1,164_ = 43.70, *P* < 0.0001; cross type × sex: F_1,164_ = 3.621, *P* = 0.06; spine: cross type: F_1,2_ = 0.878, *P* = 0.448; sex: F_1,119_ = 29.01, *P* < 0.0001, cross type × sex: F_1,119_ = 0.251, *P* = 0.617; Figure 3). Over all cross types, males consistently displayed more intense red throat and spine coloration than females (throat: F_1,712_ = 144.62, *P* < 0.0001; spine: F_1,631_ = 56.41, *P* < 0.0001), and a significant interaction revealed differences in color variation between the sexes across cross type (cross type × sex: throat: F_3,713_ = 4.77, *P* = 0.027; spine: F_1,631_ = 12.691, *P* < 0.0001). Using mixed models and *post hoc* comparisons, we tested each sex for differences in throat and spine coloration between MatLab, F1 hybrids, and backcross. Among females, backcross females had more intense red color than F1 hybrids, but were not significantly different from MatLab (F_2,4_ = 8.35, *P* = 0.037; pairwise comparisons: backcross and F1, *P*_FDR_ = 0.031; MatLab and backcross, *P*_FDR_ = 0.263). There were no significant differences for males (*P* = 0.195). Although backcross females display substantial variation in spine coloration, they did not differ relative to F1 or MatLab females (*P* = 0.212), even after controlling for family effect. The backcross and F1 males did not differ significantly for spine coloration (*P* = 0.06).

**Figure 4:**
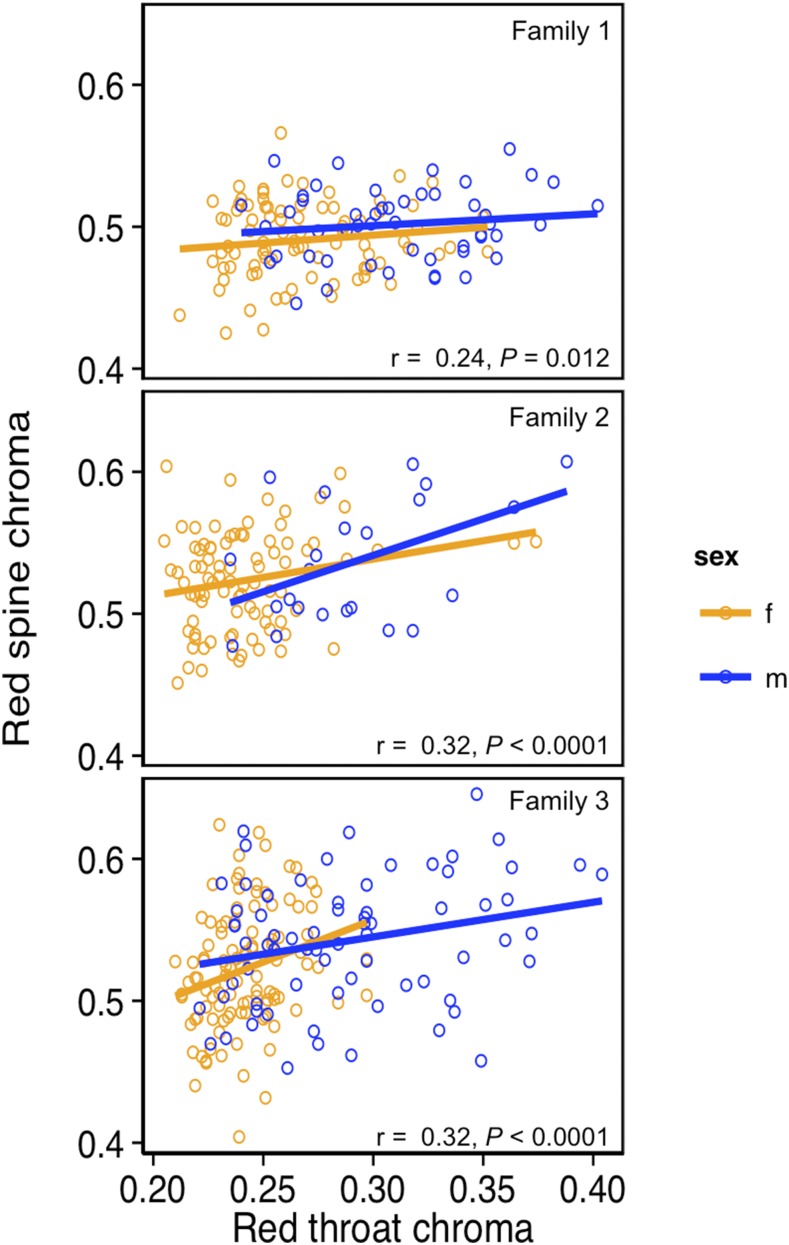
Relationship between throat and spine chroma in each half-sib backcross family (1–3). Sample sizes for each sex within each backcross family are as follows: (1) m = 51, f = 86; (2) m = 24, f = 87; (3) m = 73, f = 108.

In the backcross, spine chroma was significantly associated with throat chroma, with no differences between the sexes (throat chroma: F_1, 419_ = 35.96, *P* *<* 0.0001; sex: F_1,419_ = 1.032, *P* = 0.310; throat chroma × sex: F_1, 419_ = 0.165, *P* = 0.685). Individual analyses of each half-sib backcross revealed that red throat chroma was correlated with spine chroma (*r* = 0.21–0.32, *P* ≤ 0.012 for all families; Figure 4) in both males and females (pooled since there were no differences between the sexes), suggesting a shared mechanism influences color variation in the two ornaments.

### Three QTL contribute to red throat color, and one QTL is shared between spine and throat chroma

**Figure 5:**
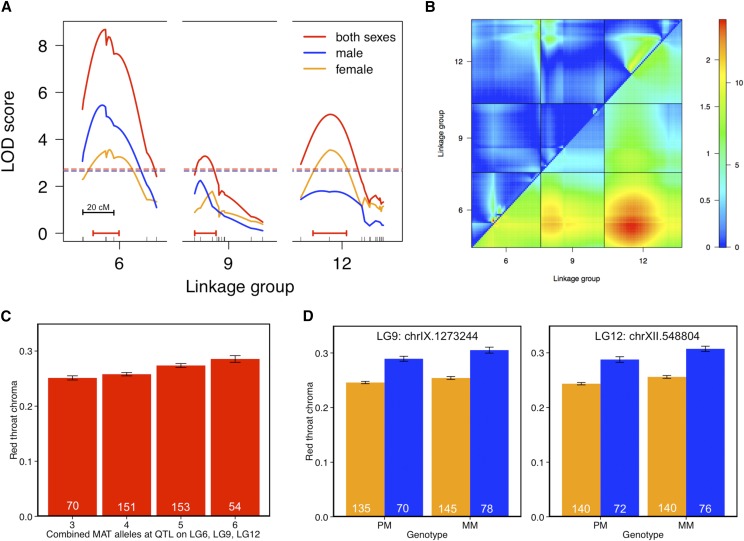
QTL analysis of red throat chroma in the backcross with both sexes included (red), males only (blue), and females only (orange). (A) Linkage groups (LG) 6, 9, and 12 contain QTL associated with throat chroma (log-transformed) in both males and females. Dotted lines represent LOD significance threshold (genome-wide α = 0.05, 1000 permutations) for each analysis (*i.e.*, both sexes, males, or females). Horizontal red lines correspond to 95% Bayesian confidence intervals for both sexes. Genetic markers are shown as tick marks on the *x*-axes. (B) Two-dimensional genome scan between significant QTL on LG 6, 9, and 12. The upper left hand triangle represents test for epistasis, whereas the lower right tests for additivity among loci. The color scale on the right indicates separate LOD score scales (for both scales, significance threshold is 4.7) for epistasis (left) and additivity (right). (C) Combined effects of MAT alleles (both sexes included) across significant QTL on red throat chroma, where an increase in the number of MAT alleles enhance red throat coloration. (D) Genotype-phenotype association analysis at the genetic marker closest to the QTL peak for each sex; the phenotypic values of red throat chroma (mean ± SE) are indicated for each genotype. MM, Matadero/Matadero; PM, Paxton/Matadero.

To characterize the genomic locations and their effect size for both ornaments, we first performed QTL mapping on all individuals, *i.e.*, including both nesting males and females. We found three unlinked QTL on autosomes (LG6, LG9, LG12), which together explained approximately 8% of variation in throat chroma (Figure 5A and Table 1). The direction of the phenotypic effects at each of the QTL was consistent with our expectation, such that fish with Matadero alleles (MAT/MAT) had elevated throat chroma relative to those with a heterozygous genotype (MAT/PAX) (Table 1). While there were no epistatic interactions between QTL, we found evidence for significant additive effects across the three QTL on throat coloration such that substitution of a MAT allele at any of the QTL caused an increase in throat chroma (Figure 5, B and C).

**Table 1:** Genome wide significant QTL for red throat and spine chroma using both sexes in the analysis

Trait	LG	Map Position (cM)	Nearest Marker (Chromosome: Position in bp)	LOD	PVE	PM	MM
Red throat chroma	6	15	chrVI.6312798	8.68	4.62	0.2603 (± 0.003)	0.2752 (± 0.003)
	9	7	chrIX.1273244	3.29	1.33	0.2616 (± 0.003)	0.2737 (± 0.003)
	12	19	chrXII.548804	5.06	2.95	0.2611 (± 0.003)	0.2744 (± 0.003)
Red spine chroma	6	7	chrVI.657036	5.76	6.31	0.5088 (± 0.003)	0.5289 (± 0.003)

For each QTL, the linkage group (LG), genetic map position (cM), nearest marker next to the highest LOD score (likelihood of odds), and percentage variance explained (PVE) are provided. The phenotypic values of each trait (mean ± SE) for each genotype (PM = Paxton/Matadero; MM = Matadero/Matadero) at the nearest genetic marker.

To test whether the three identified QTL contribute to sex-specific throat coloration, we conducted separate QTL analyses for each sex. In females, QTL on LG6 and LG12 remained statistically significant at a genome-wide threshold level, whereas only the QTL on LG6 was significant in males (Figure 5A). The QTL for throat coloration on LG9 and LG12 did not consistently reach the genome-wide threshold significance in the sex-specific QTL analyses, likely due to the lower sample size in each group. Thus, we also conducted QTL-specific tests for associations between red throat chroma and individual markers on LG9 and LG12 (*i.e.*, specific significance tests for those QTL, not genome-wide tests), and found that males and females with MAT alleles at both loci had more intense red throat chroma (LG9:chrIX.1273244: males: F_1,143_ = 10.48, *P* = 0.0015; females: F_1,276_ = 3.92, *P* = 0.048; LG12:chrXII.548804: males: F_1,143_ = 7.26, *P* = 0.008; females: F_1,276_ = 15.19, *P* = 0.0001, Figure 5D), suggesting that QTL on LG9 and LG12 do contribute to red throat coloration both in males and females. While the main analyses were conducted on post-nesting coloration, it is worth noting that single marker tests revealed that the QTL on LG 6 and LG9, but not LG12, also affected pre-nesting throat coloration in all males (LG6:chrVI.6312798: F_1,189_ = 5.02, *P* = 0.03; LG9:chrIX.1273244: F_1,189_ = 9.62, *P* = 0.002; LG12:chrXII.548804: F_1,189_ = 2.50, *P* = 0.11).

**Figure 6:**
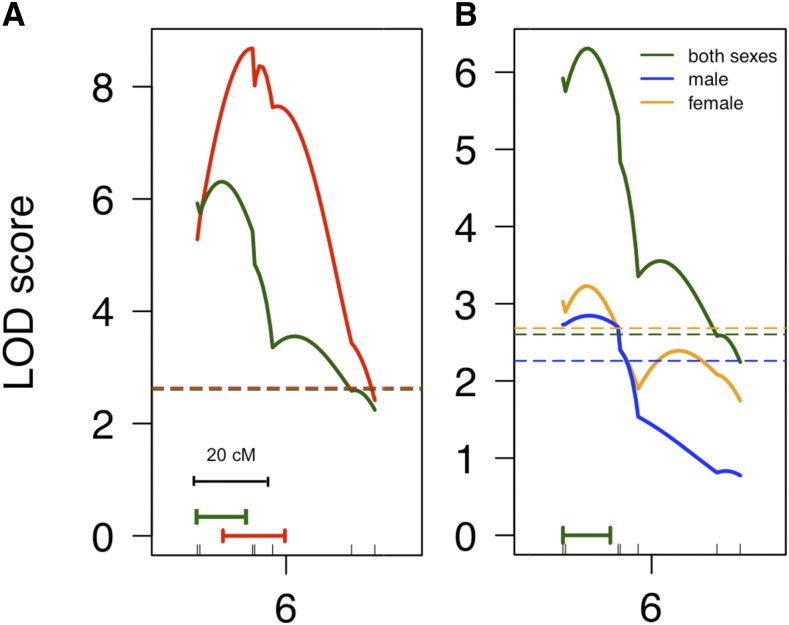
QTL analysis of spine chroma in the backcross. (A) Linkage group (LG) 6 contains QTL associated with spine chroma (green line) and throat chroma (red line). (B) Sex specific QTL analysis of spine chroma with both sexes included (green), males only (blue), and females only (orange). Dotted lines represent LOD significance threshold (genome-wide α = 0.05, 1000 permutations) for each analysis (*i.e.*, both sexes, males, or females). Horizontal green and red lines correspond to 95% Bayesian confidence intervals for spine and throat chroma, respectively. Genetic markers are shown as tick marks on the *x*-axes.

For pelvic spine coloration, we found one significant QTL, which overlapped the QTL for throat coloration on LG6 (Figure 6A). Spine coloration mapped to LG6 both in males and females (Figure 6B). Single marker analysis revealed that the QTL on LG9 (F_1,421_ = 5.29, *P* = 0.02), but not on LG12 (F_1,421_ = 0.001, *P* = 0.976), also had an effect on spine chroma, although this result was not significant at the genome wide level. Altogether, our results suggest that males and females share a similar genetic architecture for both red spine and throat chroma.

### Candidate genes related to pigment production and chromatophore cell type development are found within the QTL regions

We scanned for putative candidate genes that might be related to red chroma within the QTL regions. Reasonable candidate genes were determined based on whether they were related to processes in pigment production and pigment cell differentiation, and are listed in Table 2.

**Table 2:** List of candidate genes located in the 95% Bayesian credible intervals

LG	Nearest Marker	Gene Name	Gene Location	Inferred Gene Function	Reference
6	chrVI.657036	*Pcbd1*	2860444	Pteridine Synthesis	Verri *et al.* (2012)
6	chrVI.6312798	*Slc2a15a*	3828235	Pigment cell development	Kimura *et al.* (2014)
9	chrIX.2089567	*Slc24a3*	2277784	Pigment cell development	Verri *et al.* (2012)
9	chrIX.803523	*Sox10*	839980	Pigment cell development	Dutton *et al.* (2001)
12	chrXII.548804	*Csf1*	720731	Pigment cell development	Patterson and Parichy (2013)

The nearest marker represents the SNP marker on the genetic linkage map closest to the candidate gene. The gene location is the position in base pairs of the candidate gene on the associated chromosome.

## Discussion

The present study demonstrates that variation in red throat coloration in both females and males maps to three common autosomal regions (LG6, LG9, and LG12) in our threespine stickleback population. Consistent with the observation that red coloration is not sex-limited or restricted to males, autosomal loci are facilitating the expression of the male-typical trait in female sticklebacks, which might explain how this trait can evolve in females (Lande 1980; Lindholm and Breden 2002; Mank 2009). An overlapping QTL region on LG6 that underlies throat color contributes to red spine coloration in both sexes, suggesting that common genetic mechanisms may control both throat and spine coloration. Our findings are consistent with previous empirical studies that have shown a common genetic architecture between the sexes provides the raw material for the expression of male-typical colorful traits in females, and the evolution of mutually shared ornaments (Lande 1980; Wright *et al.* 2008; Schielzeth *et al.* 2012; Kraaijeveld 2014).

While a genetic correlation between the sexes is in some respects the default explanation for the evolution of characters such as red throats in females (Lande 1980), the mechanisms and selective pressures driving variation in sexual dimorphism in such traits are still unclear. To date, there is little evidence that direct selection contributes to the evolution of throat coloration in females. In our laboratory experiments, we have found that the female red throat does not necessarily offer a competitive or courtship advantage relative to duller throat color (Wright *et al.* 2015; Yong *et al.* 2015). At the same time, we have found little evidence that females with red throats incur fitness costs (Yong *et al.* 2013), suggesting that female throat coloration could be a neutral trait, or nearly so. One possible and unexplored explanation for the appearance of red throats in female sticklebacks is relaxed selection in the stream population (Lahti *et al.* 2009). For example, relaxed selection, in the form of reduced fish predation, is thought to have influenced lateral plate loss or reduction in lacustrine stickleback populations (Reimchen 1992, 1995). A similar lack of selective pressure might have allowed the female trait to evolve in streams. This could have been mediated via changes in genetic loci that normally repress ornament expression specifically in females. A somewhat analogous example is found in *Drosophila melanogaster*, in which inhibition of the pigment-repressing gene, *bric-a-brac* (*bab*), facilitates the expression of dark male-typical pigmentation in females (Kopp *et al.* 2000). We hypothesize that key genes residing within identified QTL regions might provide a similar mechanism for throat color expression in both males and females. Alternatively, different mechanisms that are not sex-specific might also be involved. It is possible that more efficient uptake, or differences in allocation, of red-based carotenoids might be occurring in stream sticklebacks, thereby facilitating the red color expression, as found in some salmonid species (Craig and Foote 2001). Because carotenoids can be physiologically beneficial in females, selection for mechanisms related to increased carotenoid uptake might also be possible in our stream population. Collecting more information about selection on female coloration in nature will be essential to elucidate how selection has shaped male-typical throat color expression in some populations but not others.

Some aspects of our experimental design and results call for cautious interpretation. Due to the nature of our backcross experimental design, we did not attempt to ascertain the potential contribution of the X chromosome to female red throat color, mainly because the X-chromosome did not undergo recombination with the Y-chromosome in our F1 male, except within the pseudoautosomal region (Peichel *et al.* 2004; Ross and Peichel 2008; Broman and Sen 2009; Roesti *et al.* 2013). Since all of the alleles on the X-chromosome originated from the MAT population in our backcross, we were unable to associate any segregating variants between MAT and PX with differences in red coloration. Thus, unstudied loci on the X could contribute to throat coloration. Also, while we detected three significant QTL using the whole dataset (429 backcross individuals), those on LG9 and LG12 were no longer significant at a genome-wide threshold level when each sex was analyzed separately. This is probably due to the modest size of the two samples, which could have affected our ability to detect other contributing loci with even smaller effects.

In addition to investigating female red throats, our study is the first to dissect the genomic basis of pelvic spine coloration. This is novel for several reasons. First, while many studies have focused on the genetic basis for the structural evolution of the pelvic spine in sticklebacks (Cresko *et al.* 2004; Shapiro *et al.* 2004; Coyle *et al.* 2007; Chan *et al.* 2010; Shikano *et al.* 2013), only one other study has examined the genetic contribution to variation in spine coloration, despite substantial differences in coloration between populations (Nordeide 2002; Yong *et al.* 2013; Amundsen *et al.* 2015). Here, we find a QTL for spine coloration, suggesting that spine coloration in our system has a stronger heritable component than indicated by the results of Amundsen *et al.* (2015), which revealed low and nonsignificant heritability using within-population rearing experiments in Norwegian sticklebacks. Second, a shared genetic basis for both red throat and spine is consistent with pleiotropy among characters, and between the sexes (McKinnon and Pierotti 2010). Considering that both throat and spine color patches must possess some form of red-based pigments, the same genes related to pigment allocation and production may be involved (Nordeide *et al.* 2006; Pike *et al.* 2011).

Fish body coloration results from gene networks that include both the type of pigment being produced and the development of pigment cells, *i.e.*, chromatophores (Kelsh 2004). We have identified several candidate genes related to both processes within our identified QTL regions (Table 2). For example, the largest QTL on LG6 harbors the gene *pcbd1*, which codes for a protein involved in the synthesis of pteridine pigments (Braasch *et al.* 2007). Pteridines share many of the spectral and chemical properties of carotenoids, and are responsible for the observed yellow-orange coloration in the xanthophores of fishes, such as guppies and killifish (Grether *et al.* 2004; Johnson and Fuller 2015). Carotenoids are suggested to be the main pigments for the red throats of stickleback (Wedekind *et al.* 1998; Pike *et al.* 2011). Thus, the suggestion, even if indirect, that another pigment class such as pteridines might also be responsible for the red coloration is surprising, especially considering that a previous study suggests no evidence for pteridines in the skin of a landlocked freshwater stickleback population (Nordeide 2002). However, only small regions around the pelvic spine were sampled in that study, and no chromatographic analyses were conducted. Unlike carotenoids, pteridine pigments can be synthesized *de novo* from carbohydrates and proteins, and thus might be less costly to produce (Grether *et al.* 2004). Further comparative biochemical assays would be essential to validate whether pteridines are involved in pigmentation in the MAT population.

Genes related to pigment cell development are also found in the three QTL regions. The *Sox10* gene is found on LG9 and encodes a transcription factor implicated in cell differentiation during neural crest specification (Braasch *et al.* 2007). In *Sox10* null mutants in zebrafish, there is a lack of xanthophore development (Dutton *et al.* 2001). Similarly, a colony stimulating factor (*csf1*) gene important for xanthophore development is located on LG12; overexpression of this gene results in increased xanthophore density in zebrafish (Patterson and Parichy 2013). Other candidate genes found in our QTL regions include solute transporter carrier genes (*Scl*), which have been implicated in many biological functions in fishes (reviewed in Verri *et al.* 2012), including pigmentation (Lamason *et al.* 2005). In our study, we find the *Slc2a15a* gene in the QTL on LG6, which is involved in xanthophore cell differentiation in *Oryzias* *latipes* (Kimura *et al.* 2014). The *Slc24a3* gene on LG9 is involved in the transport of cations and anions across membranes (Verri *et al.* 2012). While little is known about *Slc24a3*, it is possible that its function is similar to that of *Slc24a5*, whose functions broadly include the regulation of variation in melanin levels in both mammals and fish (Lamason *et al.* 2005; Verri *et al.* 2012). Because developmental trade-offs between melanophores and xanthophores can occur (O’Quin *et al.* 2012), it is possible that such genes might mediate cell-lineage determination early in development. Clearly, body pigmentation arises from a complex genetic network involved in both cell-type development and pigment production, and identifying the responsible genes and mutations will be a challenge. Still, our results should serve as an important guide for testing candidate or novel genes within the mapped intervals. Approaching those genes within an evolutionary developmental framework, as has been done in other fishes (Parichy and Spiewak 2015), should be an important complementary step for fully appreciating how genes and gene networks interact to produce body color variation in vertebrates.

### Conclusions

Recent years have seen advances in the genomic studies of secondary sexual characters (Kraaijeveld 2014; Johnsson 2015), and our investigation of the genetic basis of variation in throat and spine color contributes to the growing field. Our work provides a foundation for future genetic studies, and complements quantitative genetic and theoretical approaches to studying secondary sexual characters (Wilkinson *et al.* 2015). While there has been considerable investigation of male sexual characteristics in threespine sticklebacks, ours is one of the first to delve further into the genetics of sexual characteristics in females. We provide evidence that a male-typical sexually selected trait in females results in part from genomic regions sharing a similar function in males, and that pleiotropy might mediate the coloration of both throat and spine color patches. Due to the limited function for the female red throat in the context of social interaction and selection, and the shared architecture with males, the female ornament may be a correlated byproduct in the derived stream population, perhaps with little cost to females. This unusual stickleback population should offer excellent opportunities for dissecting the detailed genetic mechanisms underlying the evolution of sexual color ornaments and elucidating their sex-specific regulation. With rapid technological advances in genomics, sticklebacks may prove to be a model not only for the study of ecological speciation, but also the genomic study of sexual selection and transitions in vertebrate sexual dimorphism.

## Supplementary Material

Supporting Information
