# Supplementary material for: Genetic Architecture of Conspicuous Red Ornaments in Female Threespine Stickleback
Source: G3 (Bethesda). 2015 Dec 29;6(3):579–88. doi: 10.1534/g3.115.024505 (PMC4777121; doi:10.1534/g3.115.024505)
Supplement: Supporting Information [file supp_6_3_579__index.html]

Genetic Architecture of Conspicuous Red Ornaments in Female Threespine Stickleback — Supporting Information 

# Genetic Architecture of Conspicuous Red Ornaments in Female Threespine Stickleback

## Supporting Information for Yong, Peichel, and McKinnon, 2016

**Files in this Data Supplement:**

- Table S1 - List of the 229 single nucleotide polymorphism (SNPs) used for QTL mapping. (.pdf, 260 KB)
- File S1 - Genotype and phenotype data used for the backcrosses (need to be formatted for r/qtl). (.xls, 1,424 KB)
