# Supplementary material for: Genetic Architecture of Conspicuous Red Ornaments in Female Threespine Stickleback
Source: G3 (Bethesda). 2015 Dec 29;6(3):579–88. doi: 10.1534/g3.115.024505 (PMC4777121; doi:10.1534/g3.115.024505)
Supplement: Supporting Information [file supp_g3.115.024505_TableS1.pdf]

Table S1: List of the 229 single nucleotide polymorphism (SNPs) used for QTL mapping

Linkage Group (LG)	Map Position (cM)	SNP Marker Name (Chromosome: Position in bp)	NCBI Assay ID ss#
1	0	chrUn:37631434	244223001
1	11.513	chrI:1245655	418641981
1	26.066	chrI:1549902	244222767
1	50.083	chrI:3310077	244222768
1	53.098	chrI:4219350	244222770
1	55.76	chrI:25560380	418642013
1	55.761	chrI:26879230	244222777
1	55.763	chrI:15145305	418642000
1	56.2	chrI:23569502	244222776
1	56.631	chrI:22361077	120258417
1	58.2	chrI:21487034	418642008
1	66.029	chrI:20584613	418642006
1	123.738	chrI:27642534	418642015
2	0	chrII:4530808	120258423
2	0.441	chrII:1388455	120258419
2	1.495	chrII:2037585	418642023
2	1.495	chrII:533883	120258418
2	1.495	chrII:5914538	418642030
2	3.292	chrII:10092618	418642034
2	3.763	chrII:13353603	244222783
2	19.769	chrII:19985741	244222785
2	40.546	chrII:21231538	244222786
2	70.033	chrII:22644752	418642054
2	83.216	chrII:3931852	418642025
3	0	chrIII:639237	418642059
3	0.466	chrIII:706165	244222789
3	9.963	chrIII:11302839	418642071
3	10.45	chrIII:12316694	252841106
3	10.945	chrIII:12433574	418642074
3	12.259	chrIII:13520975	252841102
3	13.151	chrIII:13929118	244222792
3	14.949	chrIII:14393183	418642084
3	15.122	chrIII:14456990	252841063
3	35.388	chrIII:15793968	418642089
3	42.251	chrIII:16224572	120258430
3	42.481	chrIII:16251071	120258431
3	49.065	chrIII:16463929	244222796
4	0	chrIV:1738651	418642098

4	53.826	chrIV:8576794	244223068
4	54.051	chrIV:10812344	244223042
4	54.836	chrIV:13850026	244223049
4	55.407	chrIV:29763654	120258443
4	57.267	chrIV:30568387	252841083
4	63.265	chrIV:31350187	418642140
4	66	chrIV:31486885	418642142
4	67.225	chrIV:31611147	252841084
4	69.958	chrIV:31740478	244222809
4	75.256	chrIV:32033500	244222810
4	76.772	chrIV:32092919	252841132
4	78.251	chrIV:32277841	418642146
4	97.632	chrUn:27589750	418642633
4	100.686	chrUn:27402745	252841068
5	0	chrUn:11980918	252841136
5	10.783	chrV:8327818	244222816
5	11.011	chrV:8562218	244222817
5	13.564	chrV:9092208	120258450
5	16.189	chrV:9911653	418642165
5	37.135	chrV:11642284	418642179
5	39.114	chrV:11542501	120258452
5	53.601	chrUn:25831365	418642629
6	0	chrUn:15052344	418642594
6	0.692	chrVI:657036	244222822
6	15.132	chrVI:6312798	418642187
6	15.721	chrVI:7249692	418642188
6	20.638	chrVI:11954719	418642192
6	42.27	chrVI:15390272	244222831
6	48.652	chrVI:15780594	244222833
7	0	chrVII:286225	252841088
7	38.508	chrVII:1521362	120258455
7	44.755	chrVII:2559099	418642220
7	53.532	chrVII:5936068	120258457
7	53.535	chrVII:13205977	252841062
7	53.535	chrVII:16848769	418642232
7	53.535	chrVII:18353106	244222839
7	53.764	chrVII:21369768	418642239
7	54.81	chrVII:22842571	252841120
7	54.88	chrVII:23463111	252841116
7	56.883	chrVII:24610097	252841098
7	59.161	chrVII:25193081	418642246
7	62.638	chrVII:25662266	120258460
7	65.651	chrVII:25910223	418642247

7	66.62	chrVII:25986275	418642248
7	111.372	chrVII:27918897	418642257
8	0	chrVIII:1293153	252841114
8	1.728	chrVIII:1929053	244222843
8	2.833	chrVIII:3281178	120258463
8	3.435	chrVIII:4503012	244222845
8	5.907	chrVIII:9763365	252841173
8	6.336	chrVIII:13613729	244222847
8	6.746	chrVIII:13577518	252841097
8	28.277	chrVIII:16826029	244222852
8	38.646	chrVIII:17359071	252841141
8	62.269	chrVIII:19282658	418642286
9	0	chrIX:803523	252841065
9	3.821	chrIX:1273244	244222857
9	11.564	chrIX:2089567	244222858
9	14.595	chrIX:12869521	252841147
9	15.745	chrIX:8851078	252841178
9	15.745	chrIX:8852807	244223071
9	15.967	chrIX:8586014	244223070
9	17.723	chrIX:5403530	120258474
9	19.323	chrIX:5329484	244222861
9	19.711	chrIX:4882924	120258472
9	37.077	chrIX:18826248	418642319
9	44.642	chrIX:19322448	418642320
10	0	chrUn:14127611	418642619
10	0.656	chrX:1010922	418642325
10	1.019	chrX:1245433	120258479
10	13.355	chrX:7113953	120258483
10	14.982	chrX:8703061	120258485
10	15.195	chrX:8877592	252841109
10	15.863	chrX:9396721	252841115
10	17.085	chrX:10415917	252841095
10	17.431	chrX:11139448	252841128
10	19.435	chrX:11660862	418642343
10	33.84	chrX:12844036	418642350
10	37.775	chrX:13132917	418642352
10	60.333	chrX:14265366	120258486
10	60.659	chrX:14456479	252841100
11	0	chrXI:234849	120258487
11	0.462	chrXI:1017481	120258488
11	9.359	chrXI:9039275	252841094
11	10.344	chrXI:12097498	418642375
11	14.389	chrXI:12550151	418642376

11	14.389	chrXI:12746496	244222884
11	20.065	chrXI:14286902	120258493
11	21.007	chrXI:14426451	418642377
11	39.851	chrUn:32523521	418642646
11	59.665	chrXI:16655205	120258495
12	0	chrXII:70478	418642386
12	18.693	chrXII:548804	252841119
12	40.034	chrUn:17470353	418642620
12	41.945	chrXII:1969537	252841070
12	45.189	chrXII:13045611	244222894
12	45.189	chrXII:14346080	244223084
12	45.189	chrXII:15046849	418642410
12	45.645	chrXII:11472159	418642407
12	49.129	chrXII:6745006	244222892
12	49.988	chrXII:5521301	418642402
12	50.066	chrXII:6913126	120258500
12	51.716	chrXII:5828898	418642403
12	51.89	chrXII:5094466	120258499
12	52.766	chrXII:4042442	244222891
12	53.312	chrXII:18221941	244222899
12	53.331	chrXII:2713984	418642397
12	53.331	chrXII:3026329	418642398
12	53.331	chrXII:4322414	120258498
12	54.154	chrXII:17758877	244222897
13	0	chrXIII:1001571	120258503
13	0.429	chrXIII:531198	418642419
13	1.317	chrXIII:2523163	120258505
13	2.664	chrXIII:3109522	120258506
13	12.148	chrXIII:12083700	252841126
13	29.414	chrXIII:18470329	252841124
14	0	chrUn:36334731	244223000
14	0.901	chrXIV:265589	252841057
14	3.522	chrUn:38561237	252841055
14	5.689	chrUn:35285565	418642649
14	9.524	chrXIV:348659	418642435
14	10.787	chrXIV:451065	120258511
14	23.162	chrXIV:1087388	418642439
14	30.959	chrXIV:1383447	244222908
14	33.926	chrXIV:1442872	120258512
14	37.2	chrXIV:1713227	120258513
14	53.159	chrXIV:2345947	244222909
14	60.891	chrXIV:3414352	120258514
14	61.376	chrXIV:3598443	418642452

14	66.653	chrXIV:6641188	418642455
14	67.111	chrXIV:6992838	418642456
14	69.365	chrXIV:11054767	120258517
14	69.527	chrXIV:10399121	244222910
14	69.75	chrXIV:15137805	418642462
14	70.421	chrXIV:14049917	252841090
14	70.871	chrUn:21213332	120258571
15	0	chrUn:33627890	120258574
15	15.276	chrXV:414608	120258519
15	30.093	chrXV:1902350	244222911
15	34.388	chrXV:2377782	244222913
15	47.626	chrXV:5929959	244222916
15	50.087	chrXV:7200442	244223089
15	56.188	chrXV:12281774	418642480
15	56.349	chrXV:15139567	244222918
16	0	chrXVI:9428786	244222926
16	0.828	chrXVI:13921749	120258527
16	1.227	chrXVI:14283264	244222932
16	1.5	chrXVI:14688989	252841047
16	1.501	chrXVI:14550048	252841073
16	2.078	chrXVI:14963879	244222933
16	4.816	chrXVI:18106789	120258529
16	30.753	chrXVI:16673569	120258528
17	0	chrUn:56537801	244223003
17	27.1	chrXVII:3843835	120258534
17	27.596	chrXVII:4793150	418642519
17	29.818	chrUn:498491	244222983
17	30.044	chrXVII:8770036	244222945
17	30.939	chrXVII:9697366	244222947
17	66.453	chrXVII:12666712	418642526
17	72.855	chrXVII:13795831	252841087
17	76.367	chrXVII:14127979	418642528
18	0	chrXVIII:8193702	120258541
18	4.208	chrXVIII:11765327	120258543
18	5.608	chrXVIII:12273872	252841150
18	6.187	chrXVIII:12501504	244222956
18	8.053	chrXVIII:13193140	244222957
18	26.767	chrXVIII:14415132	120258548
19	0	chrXIX:646137	252841146
19	10.117	chrXIX:897343	418641956
19	28.325	chrXIX:1472847	120258551
19	33.263	chrXIX:1546489	418641958
19	38.135	chrXIX:1689208	120258552

20	0	chrXX:1808773	418642552
20	1.474	chrXX:2885078	418642555
20	2.867	chrXX:3964789	418642556
20	5.999	chrXX:7066321	418642560
20	7.338	chrXX:12436776	252841049
20	7.338	chrXX:12810044	252841048
20	8.227	chrXX:13893619	252841139
20	8.666	chrXX:14411783	252841159
20	11.6	chrXX:16111163	418642574
20	13.121	chrXX:16137862	244222969
20	22.475	chrXX:16910805	252841165
20	30.437	chrXX:16912820	252841161
21	0	chrXXI:1893294	252841157
21	1.475	chrXXI:5791519	244222974
21	1.475	chrXXI:5793103	244222975
21	1.475	chrXXI:6037992	244222976
21	2.946	chrXXI:8268451	120258563
21b	0	chrXXI:11414383	120258567
21b	4.034	chrUn:31339987	244222998

The table presents the linkage group (LG) and map position (cM) of the 229 SNP markers used in this study. Each marker is based on the position of the SNP marker in the stickleback genome assembly (Broad S1, Feb 2006). The position of chrUn markers is based on the chrUN assembly in the UCSC genome browser. All SNP information can be queried using the NCBI assay ID (ss#) at http://www.ncbi.nlm.nih.gov/projects/SNP/
